# Derivation of Mouse Parthenogenetic Advanced Stem Cells

**DOI:** 10.3390/ijms22168976

**Published:** 2021-08-20

**Authors:** Mengyi Wei, Jindun Zhang, Jia Liu, Chaoyue Zhao, Shuo Cao, Xiaojie Yan, Baojiang Wu, Siqin Bao

**Affiliations:** 1 State Key Laboratory of Reproductive Regulation and Breeding of Grassland Livestock, Inner Mongolia University, Hohhot 010020, China; 201708015@mail.imu.edu.cn (M.W.); 21908020@mail.imu.edu.cn (J.Z.); 31508025@mail.imu.edu.cn (J.L.); 31808027@mail.imu.edu.cn (C.Z.); 31808126@mail.imu.edu.cn (S.C.); 31708142@mail.imu.edu.cn (X.Y.); wubj@imu.edu.cn (B.W.); 2 Institute for Animal Genetic Research of Mongolia Plateau, College of Life Sciences, Inner Mongolia University, Hohhot 010020, China

**Keywords:** parthenogenetic embryos, parthenogenetic diploid stem cells, epiblast stem cells, pluripotency

## Abstract

Parthenogenetic embryos have been widely studied as an effective tool related to paternal and maternal imprinting genes and reproductive problems for a long time. In this study, we established a parthenogenetic epiblast-like stem cell line through culturing parthenogenetic diploid blastocysts in a chemically defined medium containing activin A and bFGF named paAFSCs. The paAFSCs expressed pluripotent marker genes and germ-layer-related genes, as well as being alkaline-phosphatase-positive, which is similar to epiblast stem cells (EpiSCs). We previously showed that advanced embryonic stem cells (ASCs) represent hypermethylated naive pluripotent embryonic stem cells (ESCs). Here, we converted paAFSCs to ASCs by replacing bFGF with bone morphogenetic protein 4 (BMP4), CHIR99021, and leukemia inhibitory factor (LIF) in a culture medium, and we obtained parthenogenetic advanced stem cells (paASCs). The paASCs showed similar morphology with ESCs and also displayed a stronger developmental potential than paAFSCs in vivo by producing chimaeras. Our study demonstrates that maternal genes could support parthenogenetic EpiSCs derived from blastocysts and also have the potential to convert primed state paAFSCs to naive state paASCs.

## 1. Introduction

In nature, there is a mice strain LT/Sv whose oocytes can seldom spontaneously activate themselves in ovaries or oviducts, then parthenogenetic embryos are able to develop to gastrulation followed by death and the rest of the embryos form teratomas [1,2]. As parthenogenetic embryos can be obtained in vitro through incubation with 7% ethanol [3] with the expression of imprinting genes, research on genetically edited parthenogenetic embryos and monogenetic individuals has begun to emerge [4,5,6]. In 2004, Tomohiro Kono produced adult parthenogenetic mice through the fusion of fully growing oocytes and non-growing oocytes with the deletion of the *H19* gene region [4]. In 2008, Lee obtained parthenogenetic diploid stem cells from oocytes of preantral follicles using feeder, serum, and knockout serum replacement, which was able to differentiate into embryoid bodies in vitro and teratomas in vivo [7]. After ten years, Qi Zhou exhibited the generation of both bimaternal and bipaternal mice from hypomethylated haploid ESCs carrying specific imprinted region deletions, but the live bipaternal mice shortly died after birth [8]. Recently, parthenogenetic epiblast stem cells (pEpiSCs) were derived from 7.5 days post coitus (dpc) parthenogenetic implantation embryos and showed similar pluripotent gene expression and differentiation potential to those of biparental EpiSCs, though they had an unstable imprinting status [9]. The study showed the media of pEpiSCs depend on MEF, and whether pEpiSCs could convert to naïve status embryonic stem cells is still unclear. In 2009, Bao et al. obtained ESC-like cells (rESCs) from the epiblasts of gastrulas in E6.5, which suggests that the state of pluripotent stem cells depends on culture condition and even be able to progressively overcome the epigenetic barrier [10]. Furthermore, they demonstrated that pluripotent stem cells in the primed state (named AFSCs) were derived from blastocysts, and AFSCs could be converted to the naive state by changing culture conditions [11]. Thus far, whether parthenogenetic stem cells possess the ability to convert from the primed state to the naive state and whether the expression of pluripotent genes in parthenogenetic stem cells is affected during this conversion are still unknown.

Here, on the basis of our previous study [11], we first established paAFSCs in a N2B27 chemically defined medium supplemented with activin A (ActA) and basic fibroblast growth factor (bFGF) (hereinafter referred to as AF medium), and then we converted the medium by replacing bFGF with BMP4, CHIR99021, and LIF in a culture medium (henceforth referred to as ABCL medium) and obtained advanced stem cells (paASCs). The paAFSCs and paASCs showed no striking differences with EpiSCs and ASCs, respectively, regarding morphology, major pluripotency, and the expression of differentiation genes. Notably, paASCs converted from paAFSCs, had enhanced developmental potency in vivo, and contributed to E10.5 chimaeras. In this study, we provide models for the further study of the function of maternal genes in conversion between primed and naive states, and we reveal the relationship between parthenogenetic stem cells and developmental potential.

## 2. Results

### 2.1. Derivation of paAFSCs

**Figure 1:**
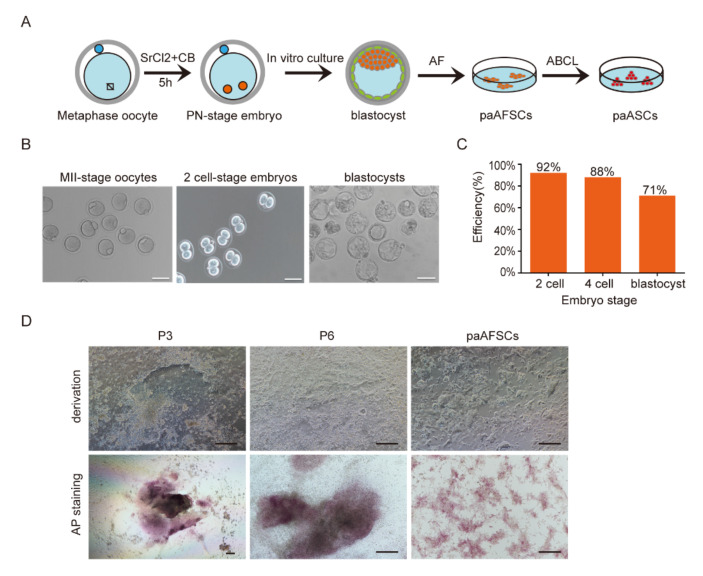
Derivation of paAFSCs from parthenogenetic diploid blastocysts: (**A**) Schematic of derivation of paAFSCs and paASCs. (**B**) The development of parthenogenetic diploid blastocysts from MII stage oocytes of ICR female mice; scale bars: 100 µm. (**C**) The developmental rates of parthenogenetic 2-cell, 4-cell, and blastocyst-stage embryos from MII oocytes of ICR female mice in vitro. (**D**) The derivation process and AP staining of paAFSCs from parthenogenetic diploid blastocysts; P3: passage 3; P6: passage 6; scale bars: 100 µm.

EpiSCs have been obtained in activated activin A and FGF signaling in vitro, but whether EpiSCs can be derived from parthenogenetic blastocysts is still unknown [3,12,13]. Thus, we attempted to establish mouse parthenogenetic diploid epiblast stem cells derived from parthenogenetic blastocysts using a chemically defined medium. First, MII oocytes from ICR female mice strain were incubated in media containing Sr^2+^ and cytochalasin B (CB) to cause the stimulation of meiosis resumption by activating a series of Ca^2+^ oscillations, which is similar to the process triggered by sperm during fertilization (Figure 1A,B) [14,15,16]. The diploid parthenogenetic embryos formed two pronuclei (PN) at a high rate and developed into two-cell stage embryos and then into blastocysts (Figure 1A,C). Next, the parthenogenetic diploid blastocysts were cultured in AF medium without either serum or feeder (a chemically defined medium) and attached to grow for 3–5 days. The derivation process of paAFSCs was found to be similar to that of EpiSCs, with the formed outgrowth clones being smooth flat, and alkaline-phosphatase-positive (AP-positive) (Figure 1A,D). The derivation efficiency of paAFSCs was 24.44% (Table 1), and paAFSCs maintained a stable morphology in AF medium over 30 passages (Figure 1D). These findings suggest that we obtained a parthenogenetic diploid stem cell line from blastocysts (paAFSCs) that shares a similar morphology and derivation efficiency with EpiSCs [12,13].

### 2.2. Characterization of paAFSCs

**Figure 2:**
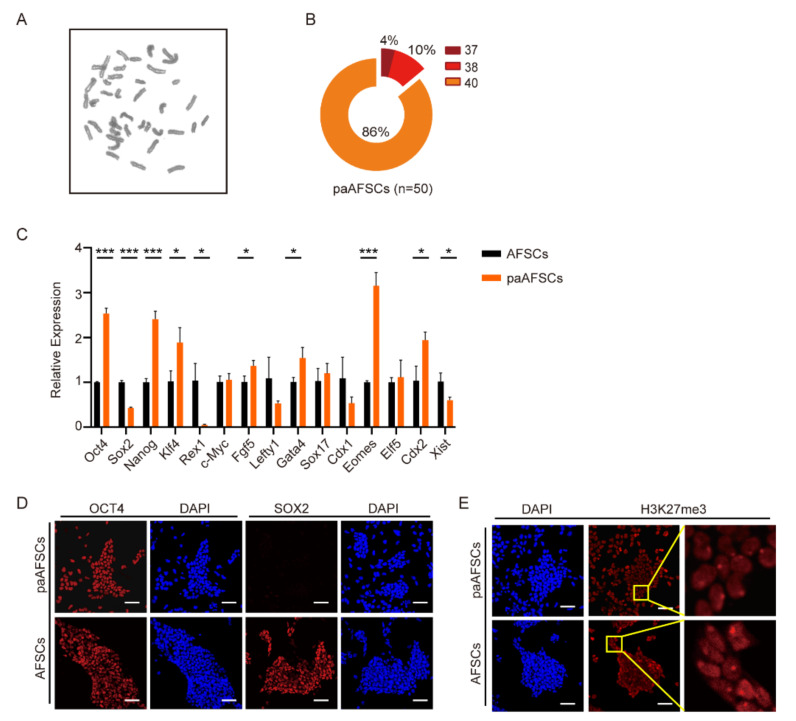
Characteristics of paAFSCs: (**A**) Karyotype of paAFSCs. (**B**) Distribution of paAFSCs with different chromosome numbers; n = 50. The cells with a normal karyotype accounted for 86% in paAFSCs. (**C**) RT-qPCR of key pluripotent genes, germ-layer-related genes, and X-chromosome-inactivation-related gene in AFSCs and paAFSCs. Error bars indicate three independent biological replicates (mean ± SD); * *p* < 0.05, *** *p* < 0.0001. (**D**) Immunostaining for pluripotent markers OCT4 and SOX2 in AFSCs and paAFSCs; scale bars: 50 µm. (**E**) Immunostaining detection of H3K27me3 foci in AFSCs and paAFSCs; scale bars: 50 µm.

To understand the properties of paAFSCs, we first observed that paAFSCs possessed normal karyotypes (Figure 2A,B). RT-qPCR results demonstrated that *Oct4*, *Nanog*, and *Klf4* had higher expression levels, but *Sox2* and *Rex1* were less expressed in paAFSCs than in AFSCs (Figure 2C) [11]. Genes related to differentiation such as *Gata4*, *Cdx2*, *Sox17*, and *Eomes* were found to be significantly more expressed in paAFSCs than in AFSCs, but *Elf5* showed similar expression levels between paAFSCs and AFSCs (Figure 2C). Another paAFSCs cell line derived from mouse strain of 129/Sv also exhibited similar RT-qPCR results (Figure S1A). Furthermore, we performed immunofluorescence and found that paAFSCs expressed main pluripotency marker OCT4 but significantly lacked SOX2, which was in line with the results presented by the RT-qPCR (Figure 2D). We also explored H3K27me3, which represents a “spot” as a distinctive mark of an inactive X chromosome through a key epigenetic modification, and the bright H3K27me3 foci with high level of *Xist* expression revealed that one of X chromosomes in paAFSCs was inactivated, as in AFSCs and EpiSCs (Figure 2E). These results indicate that parthenogenetic blastocysts have the ability to establish paAFSCs with one inactive X chromosome and maintain self-renewal in a chemically defined medium. Next, we attempted to explore the conversion ability of paAFSCs by converting AF medium to ABCL medium.

### 2.3. Derivation and Characterization of paASCs

**Figure 3:**
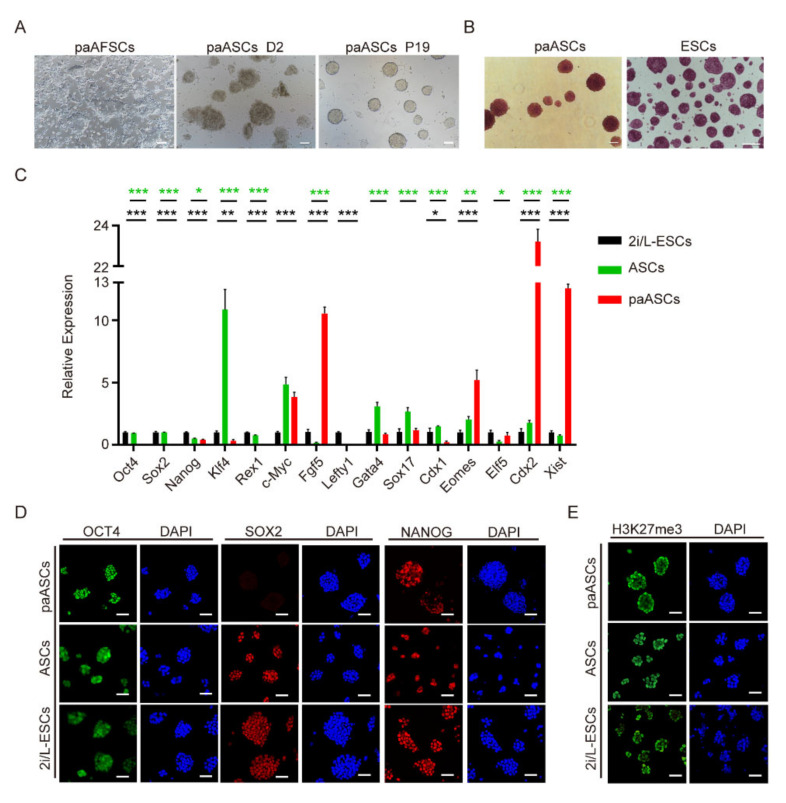
Conversion and characteristics of paASCs. (**A**) The morphological change during the conversion from paAFSCs to paASCs; D2: culture in ABCL medium for 2 days; P19: passage 19; scale bars: 100 µm. (**B**) PaASCs exhibited similar AP staining results to those of ESCs; scale bars: 100 µm. (**C**) RT-qPCR of key pluripotent genes, germ-layer-related genes, and X-chromosome-inactivation-related genes in 2i/L-ESCs, ASCs and paASCs. Error bars indicate three independent biological replicates (mean ± SD). * *p* < 0.05, ** *p* < 0.001, *** *p* < 0.0001. (**D**) Immunostaining for pluripotent markers OCT4, SOX2 and NANOG in 2i/L-ESCs, ASCs, and paASCs; scale bars: 50 µm. (**E**) Immunostaining for H3K27me3 in 2i/L-ESCs, ASCs, and paASCs. Scale bars: 50 µm.

BMP4 is known as a transcription factor secreted by primitive endoderm that affects inner cell mass development and suppresses ectoderm differentiation through Smad4 and Id1 [17,18]. CHIR99021 as a small chemical factor that stimulates canonical Wnt signal to sustain pluripotency in ESCs, and it was also mentioned as one of the two inhibitors in ‘2i’ ESCs culture medium proposed by Qilong Ying [19]. LIF was discovered in mouse ESCs medium by Austin Smith in 1987 and applied to sustain the pluripotency of ESCs [20,21,22]. To explore the impact of BMP4, Wnt, and LIF signaling on paAFSCs, we replaced the bFGF of the AF medium with BMP4, CHIR99021, and LIF (ABCL medium) (Figure 3A), and then we cultured paAFSCs. A mass of paAFSCs apoptosis and several paAFSCs transformed into domed colonies from flat colonies in the ABCL medium over 2–3 days. We collected the domed colonies and named them paASCs. Similar to ESCs, paASCs were able to self-renew for more than 30 passages and showed stronger positive alkaline phosphatase levels (Figure 3B). RT-qPCR results showed lower expression levels of *Oct4*, *Nanog*, *Sox2*, *Rex1*, and *Klf4* in paASCs than in ESCs and ASCs, but *Fgf5, c-Myc, Eomes,* and *Xist* expression increased in paASCs (Figure 3C). Interestingly, we found a significantly high expression of *Cdx2* in paASCs, which indicated that paternal genes may play important roles in impression of *Cdx2* in conversion process (Figure 3C). Immunofluorescence results demonstrated that OCT4 and NANOG were equivalently expressed in paASCs, ASCs, and ESCs, though the paASCs lacked the expression of SOX2 (Figure 3D). We further observed CDX2 expression in paASCs but not in 2i/L-ESCs, and we also found a few paASCs that exhibited both NANOG and CDX2 expression (Figure S1B). We observed no bright H3K27me3 accumulation foci in paASCs, indicating that the inactive X chromosome was reactivated during the conversion from paAFSCs to paASCs, and *Xist* exhibited a higher expression level in paASCs than in ESCs and ASCs (Figure 3C,E). Subsequently, the bisulfite DNA sequencing analysis of the maternally imprinted genes *Peg1* and *Peg3* was performed in 2i/L-ESCs, paAFSCs, and paASCs (Figure S1C). *Peg1* and *Peg3* differentially methylated regions (DMRs) were almost completely in both paAFSCs and paASCs, but 2i/L-ESCs displayed a differentially methylated pattern. These results suggest that different with 2i/L-ESCs, both paAFSCs and paASCs maintained hypermethylation during the conversion, which is different with the results of a previous study [9]. These results indicate that the maternally imprinting genes expression pattern in parthenogenetic stem cells is not affected by the conversion from primed state to naive state.

### 2.4. The Developmental Potential of paAFSCs and paASCs

**Figure 4:**
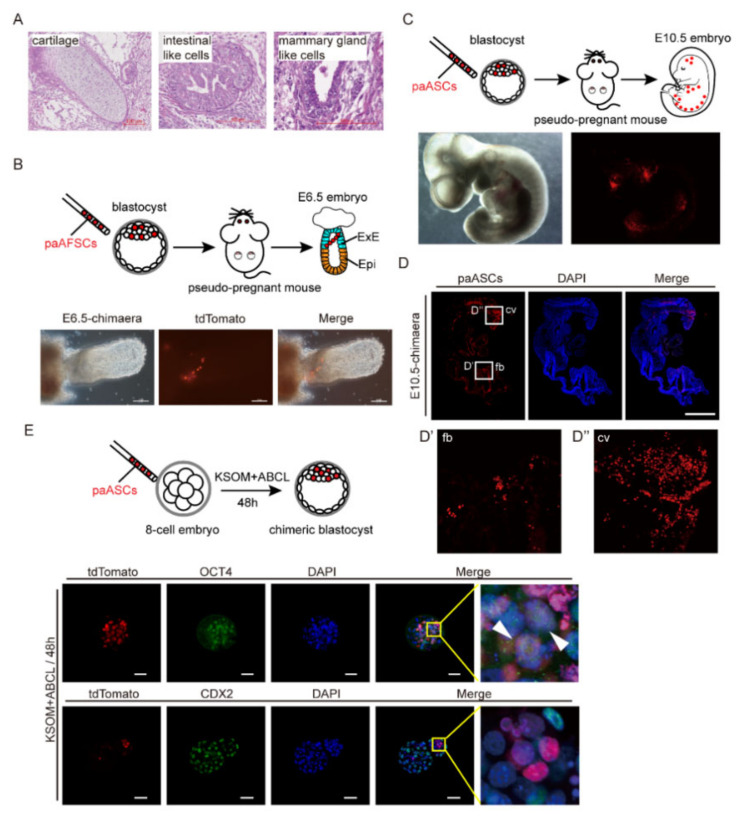
Developmental potential of paAFSCs and paASCs in vivo. (**A**) Teratoma derived from paASCs. The left panel shows a cartilage-like structure (mesoderm), the middle panel shows an intestinal-like structure (ectoderm), and the right panel shows a mammary gland-like structure (endoderm); scale bars: 100 µm. (**B**) The paAFSCs with tdTomato contributed to the extra-ectoderm of the E6.5 chimaera; scale bars: 100 µm. (**C**) The paASCs with tdTomato contributed to the fetus of the E10.5 chimaera. (**D**) Sections of the E10.5 chimaera were stained with the RFP expressed by paASCs. (**D′**) and (**D″**) mark the forebrain (fb) and caudal vertebra (cv) of fetus, respectively; scale bars: 1 mm. (**E**) Immunostaining for OCT4 and CDX2 of blastocysts which developed from 8-cell stage embryos with injected paASCs for 48 h of culturing in KA in vitro; scale bars: 50 µm. The white arrows pointed to the paASCs with tdTomato reporter also expressed OCT4.

To explore the developmental potency of paAFSCs and paASCs in vivo, we first performed teratoma formation test. The results showed that paASCs had the ability to differentiate into multiple tissue types of three germ layers in vivo (Figure 4A and Figure S2A), but paAFSCs failed to form teratomas. Next, to check whether paAFSCs and paASCs are chimaera-competent, we first injected 8–10 paAFSCs with the H2B tdTomato reporter into blastocysts followed by embryo transfer, and we found that they only contributed to the part of the extra ectoderm that was beyond the epiblast in the E6.5 embryo at a low rate (1/24), suggesting paAFSCs have the rare potential to develop to extraembryonic parts (Figure 4B and Table 2). Notably, the paASCs contributed to E10.5 embryonic tissue, thus showing that the features of paASCs are closely related to those of ESCs (Figure 4C,D and Table 3). Furthermore, to investigate the potential of paASCs in early-stage chimaeras, 8–10 paASCs were injected into eight-cell stage embryos and cultured in KSOM medium and a mixture of KSOM and ABCL at a ratio of 1:1 (named KA) respectively for a maximum of 48 h (Figure S2B). The results showed that the number of paASCs was significantly reduced in chimeric embryos for 48 h in KSOM compared to the embryos in KA (Figure S2B). We also found that the majority of paASCs expressed OCT4, and none expressed CDX2 in the KA-cultured chimaeras, which suggesting that paASCs did not contribute to trophectoderm. However, in the KSOM-cultured chimaeras, none of paASCs exhibited NANOG or SOX2 expression (Figure 4E and Figure S2C). Together, these results suggest that the developmental potency of paASCs in vitro depends on the culture conditions, as the KA medium was better able to sustain paASCs in chimeric embryos than the KSOM medium. Unlike AFSCs, we found that paAFSCs failed to form teratomas, and converted paASCs maintained pluripotency and contributed to teratomas formation and chimaeras.

## 3. Discussion

Embryonic stem cells representative of naive (ESCs) and primed (EpiSCs) pluripotency have been established and converted to each other [23,24,25]. The parthenogenetic pluripotency ESCs (paESCs) has been derived from blastocysts stage; parthenogenetic EpiSCs (paEpiSCs) have only been generated post-implantation, though the culture conditions used for all paESCs and paEpiSCs included serum or feeder [7,9,26,27] and there have no reports on the conversion of paEpiSCs to paESCs. The findings of this study have revealed that paAFSCs in a primed state can be derived from blastocysts in a chemically defined medium, as well as that primed paAFSCs can be converted to naive paASCs.

We derived paAFSCs from blastocysts by adding activin A and bFGF to a chemically defined medium (AF medium) and named paAFSCs. Compared to biparental AFSCs [11], paAFSCs were able to highly express *Oct4* and *Nanog* pluripotent markers but failed to form teratomas in vivo, thus showing that paternal gene deletion may play a role in the developmental potential of paAFSCs.

Next, we successfully converted paAFSCs to paASCs in a chemically defined ABCL medium, and we confirmed the pluripotency of paASCs with teratoma and chimaera tests. Similar to paAFSCs, paASCs were found to possess the expression of major pluripotency genes such as *Oct4* and *Nanog*, but *Sox2* had an apparently reduced expression in both cell lines. However, paASCs were found to have the capability to form teratomas and also contributed to E10.5 chimaeras, which confirmed their developmental pluripotency in vivo, consistent with previous studies [7,11,26,27]. Haploid embryonic stem cells from monogenetic gametes have been established as having a high expression of pluripotent genes and contributing to chimaera, though flow sorting every few passages for haploid cells is inevitable [28,29]. We obtained paASCs that were stable for over 30 passages and maintained pluripotency. Though we observed a decreased expression of *Sox2* in paASCs, *Cdx2* displayed enhanced expression. In 2016, Niu et al. found that a negative correlation between *Sox2* and *Cdx2* in human gastric intestinal metaplasia and *Sox2* knockdown triggered the promoter demethylation of *Cdx2* in human normal gastric epithelial cells [30]. However, whether the low expression of *Sox2* triggered the increased expression of *Cdx2* and whether this gene expression pattern is related to imprinting genes in paAFSCs and paASCs require further exploration. Moreover, the expression of *Sox2* in haploid stem cells is same as that in normal mouse ESCs [5,28,29,31], but no study of parthenogenetic diploid stem cells has shown the expression status of *Sox2* [7,26,27]. Thus, whether the low expression of *Sox2* is a common property of parthenogenetic diploid stem cells also needs further research.

Due to paASCs being derived from blastocysts, we transferred paASCs to an early-stage embryonic environment to test their pluripotency in vitro. Our results showed that a few of the paASCs developed in the pure embryo culture medium (KSOM) over 48 h. However, when the culture medium changed from KSOM to KA medium, the majority of paASCs were alive after 48 h. The results illustrated that the development of paASCs depends on the culture condition, and they still contributed to blastocysts in vitro, even with a lack of paternal genes. Furthermore, we found that the imprinting genes *Peg1* and *Peg3* maintained the same methylation pattern in paAFSCs and paASCs, and we demonstrated that the ABCL medium induced the pluripotency of paASCs, however, it failed to change the methylation status of the imprinting genes *Peg1* and *Peg3*. In contrast to a previous study that demonstrated that primed pluripotent stem cells have an unstable imprinting status, paAFSCs and paASCs maintained hypermethylation of maternally imprinting genes during the conversion [9]. As ASCs showed a hypermethylated genome, whether the stable methylation of maternally imprinting genes related to the ABCL culture condition still needs further investigation [11]. The methylation pattern of imprinting genes in the conversion between paAFSCs and paASCs needs further research. Moreover, paAFSCs and paASCs could be further differentiated in vitro to study the changes in methylation status of imprinting genes and explore the cause of death of parthenogenetic embryos during the implantation stage in vivo [32]. As we have shown, parthenogenetic pluripotent stem cells can also transition between different states (such as the naive and primed states), suggesting that epigenetic barriers could also be overcome with only maternal inheritance.

Taken together, the results of our study demonstrate that maternal genes without paternal gene modification can maintain the pluripotency and developmental potential of paAFSCs and paASCs, as well as support the conversion of parthenogenetic stem cells from the primed state to the naive state. Furthermore, paAFSCs and paASCs provide models for exploring imprinting gene expression patterns in the conversion of parthenogenetic stem cells between the naive and primed states.

## 4. Materials and Methods

### 4.1. Parthenogenetic Diploid Embryos Collection

ICR and 129/Sv eight-week-old female mice (Weitonglihua, Beijing, China) were super-ovulated via the injection of 5 IU of pregnant mare’s serum gonadotropin (PMSG) followed by 5 IU of human chorionic gonadotropin (HCG) over 48 h. Ovulated MII oocytes were collected 16 h after HCG injection in M2 and treated with hyaluronidase until the cumulus cells dispersed, and then activated in the activation medium for 4–6 h. The activated parthenogenetic diploid embryos were cultured in a KSOM medium (Millipore, Billerica, MA, USA) to develop to blastocysts. Activation medium containing 4.77 g/L of NaCl, 0.36 g/L of KCl, 0.161 g/L of KH_2_PO_4_, 0.291 g/L of MgSO_4_·7H_2_O, 5.847 g/L of sodium lactate, 1 g/L of glucose, 0.13 g/L of penicillin–streptomycin, 2.11 g/L of NaHCO_3_, 0.01 g/L of phenol red, 0.0297 g/L of sodium pyruvate, 2.66 g/L of SrCl_2_·6H_2_O, 0.146 g/L of glutamine, 0.04 g/L of EDTA, and 5 μg/L of cytochalasin B.

### 4.2. Derivation of paAFSCs and paASCs

Parthenogenetic diploid blastocysts were collected and zona pellucida were digested via treatment with Tyrode’s acid, and then they were cultured in a 5% AFR (activin A, bFGF, and 5% KSR in N2B27) medium. After 5–8 days, the flat colonies were cut into small pieces and transferred to a fresh 2% AFR (activin A, bFGF, and 2% KSR in N2B27) medium, and the developed colonies were cut and transferred to a 1% AFR (activin A, bFGF, and 1% KSR in N2B27) medium over 10–12 days. We twice repeated this manipulation of the colonies in the 1% AFR medium and replaced it with an AF medium. The paAFSCs were derived and could self-renew for 30 passages using Accutase at a ratio of 1:3–1:5 for two days. Next, we transferred paAFSCs from the AF medium to the ABCL medium, and we found that domed colonies appeared in 3–4 days. These colonies were picked and cut into smaller pieces using glass needle, and then they were transferred to a fresh ABCL medium. When these colonies had grown for 6–7 days, they were treated with Accutase (Gibco, Gaithersburg, MD, USA), and the resulting cells were capable of self-renewal for over 30 passages and named paASCs. The AF medium consisted of an N2B27 medium supplemented with 20 ng/μL of activin A (R&D Systems, Minneapolis, MN, USA) and 12 ng/μL of bFGF (R&D Systems). The ABCL medium consisted of an N2B27 medium with 20 ng/μL of activin A, 50 ng/μL of BMP4 (R&D Systems), 3 μM of CHIR99021, and 1000 IU/mL of LIF (Millipore). All using plates were coated by fibronectin (1 mg/mL in PBS; Millipore) at least 0.5 h before use.

### 4.3. Alkaline Phosphatase (AP) Staining

The cells were washed with 1 × PBS and fixed in 4% paraformaldehyde at room temperature for 30 min (min) followed by the addition of an AP staining solution (Sigma, Darmstadt, Germany). The AP staining solution was prepared as follows: we mixed 50 μL of sodium nitrite solution with 50 μL of an FRV-alkaline solution, placed the mixture at 37 °C for 3 min, added 2.25 mL of H_2_O and 50 μL of a naphthol-As-BI alkaline solution into the mixture, and finally incubated the staining solution with fixed cells in the dark overnight.

### 4.4. Karyotype

The cells were incubated with 0.2 µg/mL of colchicine in a culture medium for 2 h, dissociated with Accutase, and then centrifuged at 1500 r/min for 5 min to collect the cells. The cells were gently resuspended in 8 mL of 0.075 mol/L KCL (Sigma) and incubated in a 37 °C water bath kettle for 40 min for hypotonic treatment. One milliliter of stationary liquid (methanol:glacial acetic acid = 3:1) was subsequently added to the resuspended cells and gently mixed, followed by centrifuging at 1000× rpm for 10 min. After discarding the supernatant, the cells were gently mixed in 8 mL of stationary liquid and incubated in 37 °C water bath kettle for 30 min for cell fixation; this was repeated twice. We then resuspended the cells with 0.5 mL of stationary liquid and dripped the resuspended cells onto ice cold glass slides, followed by drying the glass slides for 1 h in a 70 °C drying oven. Then, the glass slides were stained with Giemsa (Sigma) for 10 min, washed with distilled water, air-dried, and analyzed by LUCIA Cytogenetics (Lucia, Praha, Czech Republic).

### 4.5. Real-Time Quantitative Polymerase Chain Reaction (RT-qPCR)

Total RNA was extracted with a RNeasy Mini Kit (Qiagen, Hilden, Germany), and cDNA was isolated with a GoScript Reverse Transcription System (Promega, Madison, WI, USA). RT-qPCR reactions were set up using the SYBR FAST Universal qPCR kit (KAPA Biosystems, Woburn, MA, USA). Relative expression values were normalized to GAPDH expression, and data were normalized with the 2^−ΔΔCt^ computing method. Each experiment was performed in technical triplicate. A list of used primers is shown in Table S1. Significance between different groups was determined using a *t*-test, * *p* < 0.05, ** *p* < 0.01, *** *p* < 0.001.

### 4.6. Immunofluorescence

The cells used for immunofluorescence assays were washed with PBS and fixed in 4% paraformaldehyde for 30 min at room temperature, and then they were permeabilized with 0.1% Triton X-100 (Sigma) and 1% BSA in PBS for 30 min. Then, the cells were incubated with primary antibody at 4 °C overnight. Subsequently, the cells were washed three times with 1% BSA and 0.1% Triton X-100 in PBS for 5 min each. Next, they were incubated with a secondary antibody for 1 h at room temperature in the dark, then washed once for 5 min in 1% BSA and 0.1% Triton X-100. The cells were then mounted in Vectashield with DAPI (Vector Laboratories, Burlingame, CA, USA). The samples were observed with a laser microscope (Nikon, Tokyo, Japan). The used anti-bodies are listed in Table S2.

### 4.7. Teratomas Formation

The paAFSCs and paASCs were disaggregated using Accutase, and 1 × 10^6^ cells were injected under the epithelia of NOD-SCID mice. Three-to-five weeks after transplantation, tumor(s) were collected, fixed with 4% paraformaldehyde, and processed for paraffin sectioning. Sections then were observed following hematoxylin and eosin staining. All animal experiments were performed in accordance with the National Research Council Guide for the Care and Use of Laboratory Animals and were approved by the Institutional Animal Care and Use Committee at Inner Mongolia University, China. The ethics approval number is “SCXK (Meng) 2020-0002”.

### 4.8. Production of Chimaera

Approximately 12–15 donor cells were injected into the cavities of ICR mice blastocysts (E3.5) using a piezo-assisted micromanipulator attached to an inverted microscope. The injected embryos were cultured in KSOM to enable the expansion of the blastocoel cavity and then transferred to the uteri of pseudopregnant ICR mice at 2.5 days post coitus (dpc). The embryos were isolated at embryonic stages E6.5 and E10.5, and their chimeric contributions were checked. In a similar manner to the injection of blastocysts, eight-cell stage embryos (E2.5) were injected with 8–10 donor cells carefully placed into the perivitelline space under the zona pellucida. The injected embryos were cultured in KSOM at 37 °C under a 5% CO_2_ atmosphere for 24 or 48 h and then transferred to the uteri of pseudopregnant ICR female mice at 2.5 dpc or fixed with 4% paraformaldehyde for 30 min, followed by immunofluorescence to check for chimeric contribution.

### 4.9. Cryosectioning and Immunofluorescence Staining

The collected E10.5 chimaeras were fixed in 4% paraformaldehyde for 2 h, washed with PBS for 5 min, and then dehydrated in 30% sucrose (Sigma) overnight at room temperature. Next, chimaeras were embedded in the mixture of a Tissue-Tek O.C.T. compound (SAKURA, Flemingweg, Alphen aan den Rijn, Netherlands) and 10% sucrose (1:1) for 30 min before being frozen by liquid nitrogen. The frozen Oct blocks were sectioned at 10 μm by CRYOSTAR NX50 (Thermo, Walldorf, Germany). First, sections were washed with PBS three times for 5 min each. Second, sections were permeabilized with 0.1% Triton X-100 (Sigma) for 30 min and blocked with 5% donkey serum and 1% BSA for 30 min. Then, sections were incubated with the first antibody in 5% donkey serum and 1% BSA overnight at 4 °C. We washed sections with PBS three times, 5 min each, followed by incubation with a second antibody in 5% donkey serum and 1% BSA for 1 h at room temperature. Finally, sections were mounted in Vectashield with DAPI (Vector Laboratories) and observed with a laser microscope (Nikon, Tokyo, Japan). The used antibodies are listed in Table S2.

### 4.10. Bisulfite Genome Sequencing

Genomic DNA was extracted with the TIANamp Genomic DNA Kit (TIANGEN Biotech, Beijing, China) and treated with the EZ DNA Methylation-GoldTM Kit (Zymo Research, Irvine, CA, USA) according to the manufacturer’s instructions. Then, PCR amplification was conducted using bisulfite PCR primers for Peg1 (sense: TAGGGGTTTGTTTGTTGTTTATTT; antisense: AACCTATAAATATCTTCCCATATTC) and Peg3 (sense: TAGGGGTTTGTTTGTTGTTTATTT; antisense: CATACTACAAACAACCAAATAACC). Reactions were conducted at 96 °C for 2 min, followed by amplification for 36 cycles at 95 °C for 1 min, 59 °C for 30 s, 72 °C for 30 s, and a final extension at 72 °C for 10 min. The amplified PCR products were verified by electrophoresis on 1% agarose gel, and then they were subcloned using a pMDTM19-T Vector Cloning Kit (Takara, Otsu, Shiga, Japan). Reconstructed plasmids were purified, and individual clones were sequenced.

## Figures and Tables

**Table 1:** The derivation rates of paAFSCs from ICR parthenogenetic diploid blastocysts.

No. of MII-Stage Oocytes	No. of Parthenogenetic Blastocysts	No. of paAFSCs Cell Lines
77	45 (58.44%)	11 (24.44%)

**Table 2:** The paAFSCs contributed to the E6.5 chimaera.

Stage of Embryo (Collected)	No. of Transferred Embryos	No. of Collected Embryos	No. of Chimaeras
E6.5	65	24 (36.9%)	1 (4.2%)

**Table 3:** The paASCs contributed to the E10.5 chimaera.

Stage of Embryo (Collected)	No. of Transferred Embryos	No. of Collected Embryos	No. of Chimaeras
E10.5	18	6 (33.3%)	2 (33.3%)

## Data Availability

Not applicable.

## Supplementary Materials

The following are available online at https://www.mdpi.com/article/10.3390/ijms22168976/s1.
