# Supplementary material for: Derivation of Mouse Parthenogenetic Advanced Stem Cells
Source: Int J Mol Sci. 2021 Aug 20;22(16):8976. doi: 10.3390/ijms22168976 (PMC8396573; doi:10.3390/ijms22168976)
Supplement: Supplementary file 1 [file ijms-22-08976-s001.zip › ijms-1305291-supplementary.pdf]

Supplementary Material

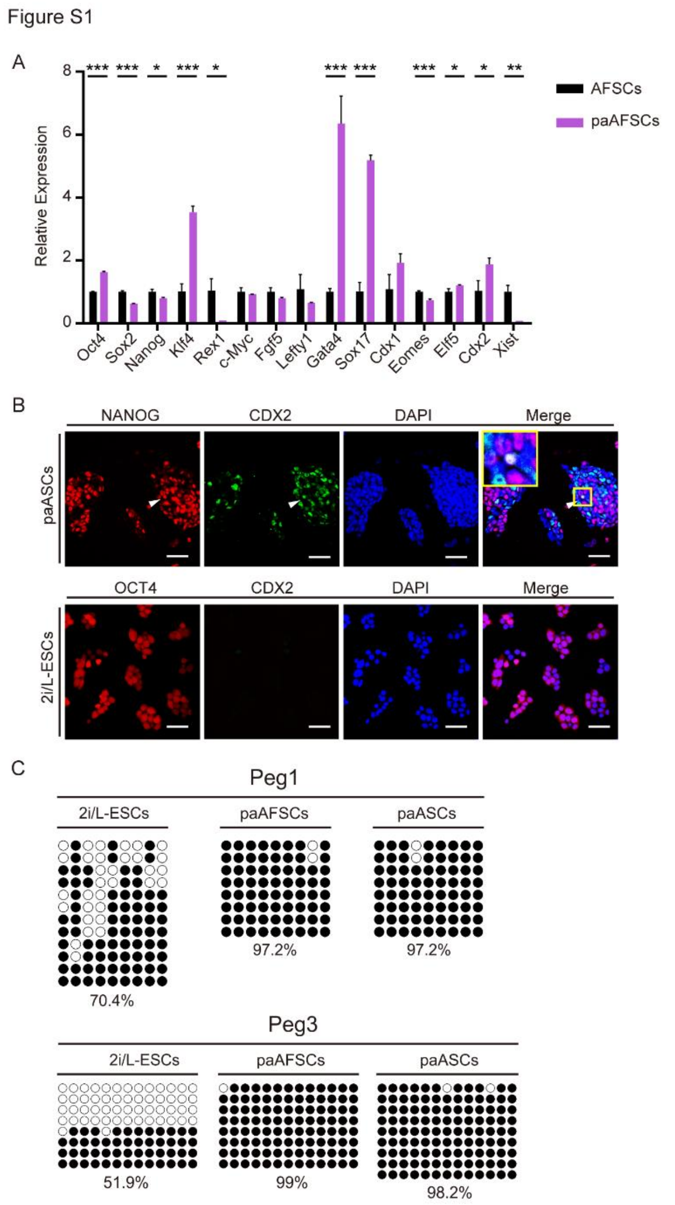

Figure S1. The characteristics of paAFSCs and paASCs. (**A**) RT-qPCR of key pluripotent genes, germ layer related genes and Xist in AFSCs and paAFSCs which derived from 129/Sv parthenogenetic diploid blastocysts. Error bars indicate three independent biological replicates (mean±SD). *$p < 0.05$, ** $p < 0.001$, *** $p < 0.0001$. (**B**) Immunostaining for NANOG and CDX2 in 2i/L-ESCs and paASCs. White arrows point to the cell both expressed NANOG and CDX2. Scale bars: 50 μm. (**C**) Methylation status of Peg1 and Peg3 in 2i/L-ESCs, paAFSCs and paASCs.

Figure S2

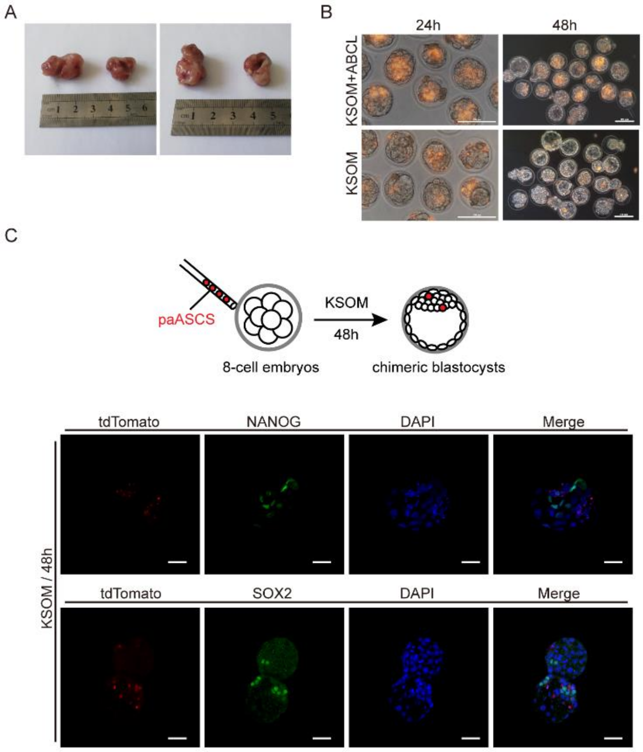

Figure S2. Development potential of paASCs. (**A**) Teratomas derived from paASCs. (**B**) The developed embryos from 8-cell embryos which injected with tdTomoto paASCs cultured for 24 h and 48 h in KSOM and KA medium. Scale bars: 100 μm. (**C**) Immunostaining of NANOG and SOX2 in blastocysts developed from 8-cell embryos which injected with tdTomoto paASCs cultured for 48 h in KSOM medium. Scale bars: 50 μm.

Table S1 Sequences of primers

**qPCR primers**		
**Gene name**	**Forward primer**	**Reverse primer**
Oct4	GCTTGGGCTAGAGAAGGATGTG	TGGCGCCGGTTACAGAAC
Sox2	CATGAGAGCAAGTACTGGCAAG	CCAACGATATCAACCTGCATGG
Nanog	AAACCAGTGGTTGAAGACTAGCAA	GGTGCTGAGCCCTTCTGAATC
Klf4	TCAAGTTCCCAGCAAGTCAG	CATCCAGTATCAGACCCCATC

Rex1	AAGCCGTATCAGTGCACGTTCGAAGGCT	ATGCGTGTATCCCCAGTGCCTCTGTCAT
C-myc	GACTCTGAAGAAGAGCAAGAAGATGA	TCCACAGACACCACATCAATTTC
Fgf5	AAACTCCATGCAAGTGCCAAAT	TCTCGGCCTGTCTTTTCAGTTC
Lefty1	TGGACAAGGCTGATGAGGAA	TGGCATGGCTGTGTTGTAGC
Gata4	TTCCTCTCCCAGGAACATCAAA	GCTGCACAACTGGGCTCTACTT
Eomes	CGGCAAAGCGGACAATAACA	GGAGCCAGTGTTAGGAGATTC
Sox17	GTCAACGCCTTCCAAGACTTG	GTAAAGGTGAAAGGCGAGGTG
Cdx1	GTAAGACCCGAACCAAGGAC	CAGGATATCCTAGGGTAGAAACTCCTCCTTGACG
Elf5	GGACCGATCTGTTCAGCAAT	GGGTGCACTGATGTCCAGTA
Cdx2	CCTGCGACAAGGGCTTGTTTAG	TCCCGACTTCCCTTCACCATAC
Xist	CTCATAGTAGTGGCCGAGTA	TAAGCCCGTTAAGTAGTCCTT
Gapdh	ACCACAGTCCATGCCATCAC	TCCACCACCCTGTTGCTGTA

Table S2 Antibodies

**Reagent**	**Source**	**Identifier**	**Dilution Ratio**
**Antibodies for IF**			
Mouse monoclonal Oct4	BD Biosciences	611203	1:200
Goat polyclonal Sox2	Santa cruz	Sc-17320	1:200
Rat monoclonal Nanog	eBioscience	14-5761	1:500
Rabbit polyclonal H3K27me3	Millipore	07-449	1:500

Mouse monoclonal Cdx2	Biogenex	Cdx2-88	1:200
Rabbit polyclonal RFP	Rockland	600-401-379	1:200
Alexa Fluor 488 donkey anti-mouse lgG	invitrogen	A21202	1:500
Alexa Fluor 488 donkey anti-goat lgG	invitrogen	A11055	1:500
Alexa Fluor 488 donkey anti-rat lgG	invitrogen	A21208	1:500
Alexa Fluor 568 donkey anti-rabbit lgG	invitrogen	A10042	1:500
Alexa Fluor 568 donkey anti-mouse lgG	invitrogen	A10037	1:500
